# Disseminated TB with central nervous system involvement

**DOI:** 10.5588/ijtldopen.25.0258

**Published:** 2025-09-10

**Authors:** S. Chi

**Affiliations:** Department of Tuberculosis, First People's Hospital of Zigong City, Zigong, China.

**Keywords:** tuberculosis, diagnosis, drug treatment, CNS-TB, China

Dear Editor

Differentiating intracranial tuberculomas and tuberculous abscesses from pyogenic abscesses and metastatic tumors is a considerable diagnostic challenge, owing to overlapping clinical and radiological presentations, including multifocal ring-enhancing lesions, perilesional edema and mass effect.^1^ Globally, there are approximately 10 million new cases of TB each year. Although central nervous system TB (CNS-TB) only makes up a small fraction of these cases, the death rate for hospitalized patients can reach as high as 42%, and about half of those who survive are left with permanent neurological problems.^2^ So, CNS-TB is not just life-threatening; it also drastically affects patients' quality of life. Countries like China and India carry a heavy burden of CNS-TB, which is evident in their significant contributions to global research on the topic, in part fueled by their high national rates of TB.^3^ Although we need more specific data on CNS-TB in China, studies in India highlight how serious the condition is. For example, data from India show that the mortality rate of CNS-TB can be as high as 35%, far exceeding that of other types of TB. In addition, it appears many cases go unreported in surveillance systems,^4^ and we therefore urgently need better ways to diagnose and manage CNS-TB. Tuberculomas can mimic high-grade gliomas or metastases on conventional magnetic resonance imaging (MRI),^5^ while tuberculous brain abscesses are often indistinguishable from those caused by bacterial or fungal pathogens.^6^ Definitive diagnosis typically requires histopathological or microbiological confirmation, procedures that may be precluded in patients deemed high-risk for surgery. This diagnostic difficulty is frequently encountered, particularly for CNS mass lesions like tuberculomas in endemic areas, often necessitating reliance on a combination of clinical findings, imaging characteristics, evidence of TB elsewhere in the body, or monitoring the response to empirical anti-TB therapy (ATT), as large cohort studies from regions like India illustrate.^7^ Against this backdrop, this case report details the successful management of disseminated TB with CNS involvement in an immunocompetent individual. The diagnosis was established through a pragmatic integration of neuroradiological findings, crucial microbiological data from a non-CNS source – i.e., bronchoalveolar lavage (BAL) – and monitoring of therapeutic response, which circumvented the need for invasive brain biopsy. The report aims to highlight the utility of this integrated diagnostic approach, particularly in scenarios where CNS lesions mimic malignancy and direct tissue sampling poses significant risks, a situation highly relevant within high-burden settings like China.

A 49-year-old, HIV-negative, immunocompetent male presented with a one-month history of dizziness, progressive right hemiparesis, cognitive impairment and cough. Initial MRI revealed multiple ring-enhancing lesions in the right cerebellum and bilateral cerebral hemispheres, suggestive of brain abscesses or metastatic disease. Thoracic imaging identified mediastinal lymphadenopathy, consolidation in the right upper lobe, and bilateral emphysema with multiple bilateral pulmonary bullae. Cerebrospinal fluid analysis performed at an external hospital revealed a chloride ion concentration of 116.7 mmol/L, a total protein concentration of 0.49 g/L, and a glucose concentration of 4.76 mmol/L. Cerebrospinal fluid analysis conducted at our institution revealed an adenosine deaminase level of 0.5 U/L, a lactate dehydrogenase level of 20 U/L, a protein concentration of 34.3 mg/dL, a glucose concentration of 5.47 mmol/L, a chloride concentration of 125.4 mmol/L, and a white blood cell count of 4×10^6^/L. CSF culture, GeneXpert, and TB DNA detection were all negative. Crucially, BAL fluid tested positive for *Mycobacterium tuberculosis* (M. tb) complex DNA via polymerase chain reaction (PCR). Despite serial neuroimaging demonstrating lesion progression and worsening edema (Figure 1), the patient declined biopsy due to perceived surgical risks. Consequently, empirical anti-TB therapy comprising isoniazid, rifampicin, pyrazinamide, and ethambutol (HRZE) was initiated. Because none of the cerebrospinal fluid parameters indicated tuberculous meningitis, corticosteroid therapy was withheld. Four months later, the patient's hemiplegia and cognitive impairment were alleviated. Head CT scan revealed patchy lesions of slightly low and low density in both cerebral hemispheres and the right cerebellar hemisphere, with surrounding edema evident in some areas. The periphery of the patchy lesion in the right temporal lobe exhibited increased density, and slight widening of adjacent cerebral sulci was observed. Consequently, the patient entered the consolidation phase and continued treatment with the HRE regimen. One year later, head CT scan revealed no significant changes in the right temporal lobe lesion and its associated edema. Although the patient's clinical symptoms, including hemiplegia and cognitive impairment, had completely resolved, the absence of significant radiological improvement in this lesion prompted the decision to extend the treatment course to prevent recurrence. After 21 months of anti-TB therapy, a follow-up head MRI scan demonstrated significant resolution of the intracranial lesions and associated edema (Figure 2).

**Figure 1.**
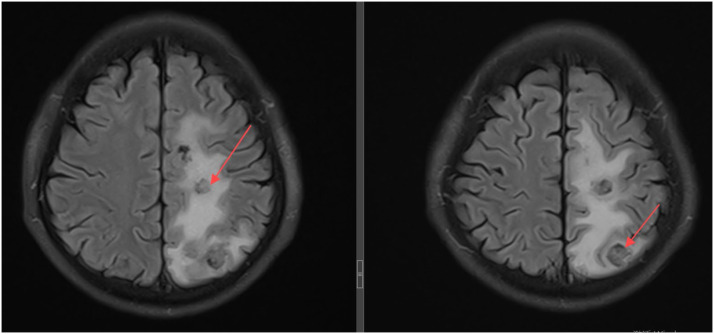
Neuroimaging demonstrating lesion progression and worsening edema. As indicated by the red arrow, prior to initiating anti-TB therapy, MRI revealed multiple ring-enhancing lesions with marked perilesional edema.

**Figure 2.**
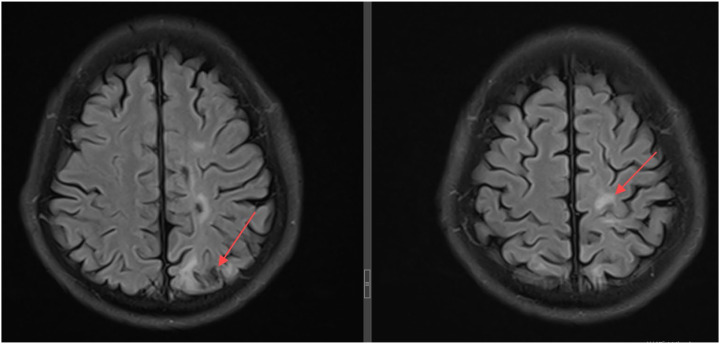
Neuroimaging demonstrating reduced edema. As indicated by the red arrow, after 21 months of anti-TB therapy, the intracranial lesion showed marked reduction in size and resolution of perilesional edema.

The neuroimaging differentiation of intracranial tuberculous lesions remains difficult due to their nonspecific features. In this patient, the multifocal enhancing lesions associated with significant perilesional edema and mass effect initially raised suspicion for metastatic disease, highlighting the diagnostic ambiguity between infectious and neoplastic processes in an immunocompetent host, a scenario that can often lower the initial index of suspicion for TB. Advanced neuroimaging techniques, such as proton MR spectroscopy (MRS) to detect lipid-lactate peaks indicative of caseous necrosis,^8^ could potentially improve diagnostic accuracy but were not available. A key aspect of this case is the pivotal role of systemic evidence in guiding the diagnosis. The presence of mediastinal lymphadenopathy and upper lobe consolidation, coupled with the definitive PCR confirmation of M.tb in BAL fluid, provided compelling indirect support for disseminated TB involving the CNS, effectively shifting the diagnostic probability away from metastatic disease or pyogenic abscesses. While histopathology remains the gold standard, the patient's marked clinical improvement and favorable radiological response to anti-TB treatment served as strong corroborative evidence. This approach, integrating non-invasive microbiological confirmation from an extracranial site with therapeutic trial monitoring, aligns with pragmatic diagnostic strategies endorsed by the WHO, particularly relevant when invasive procedures carry prohibitive risks or are declined by the patient. The successful outcome underscores the value of such an integrated pathway in resolving complex diagnostic dilemmas involving CNS lesions.

This case underscores several pertinent points relevant to the contemporary management of complex CNS-TB, particularly highlighting aspects illustrated by this specific patient's journey: 1) Demonstrating the value of empirical therapy guided by indirect evidence. In regions with high TB prevalence, or when strong suspicion arises from systemic findings, the prompt initiation of empirical anti-TB therapy can be crucial. This case illustrates how confirming extracranial TB (via BAL PCR) provided sufficient justification for empirical treatment of suspected CNS-TB, leading to a favorable outcome and averting potential neurological decline associated with diagnostic delays or the risks of biopsy. The WHO-recommended HRZE regimen demonstrated notable efficacy even without direct CNS tissue confirmation. 2) Although a 12-month regimen is often considered standard, CNS-TB complexity varies. The complete clinical and radiological resolution observed in this patient following a 21-month treatment course illustrates how extending therapy beyond the conventional duration may be beneficial in achieving optimal outcomes in select cases with severe initial presentation or slower response. However, definitive guidelines on optimal duration in such specific scenarios is evolving.^9^ 3) This report specifically emphasizes the diagnostic contribution of BAL fluid PCR (GeneXpert MTB/RIF assay in many settings) in the context of ambiguous CNS lesions. By providing definitive microbiological evidence of M.tb infection systemically, it critically informed the clinical decision-making process, strengthened the rationale for empirical anti-TB therapy, and ultimately obviated the need for a higher-risk brain biopsy.

## References

[1] Khatri GD, Magnetic resonance imaging spectrum of intracranial tubercular lesions: one disease, many faces. Pol J Radiol. 2018;83:e524-e535. 30800191 10.5114/pjr.2018.81408 PMC6384409

[2] Khellaf L, Long-term outcomes of patients with central nervous system tuberculosis in a high-income country: a retrospective study. Clin Microbiol Infect. 2025;S1198-743X(25)00089-8. 10.1016/j.cmi.2025.02.028 40024525

[3] Pant A, Global research trends in central nervous system tuberculosis - A bibliometric analysis. J Clin Tuberc Other Mycobact Dis. 2024;34:100414. 38304751 10.1016/j.jctube.2024.100414 PMC10831285

[4] Inbaraj LR, Mortality estimates of central nervous system TB in India. Int J Tuberc Lung Dis. 2023;27(11):876-877. 37880885 10.5588/ijtld.23.0308

[5] Indoria A, Radiomics features for the discrimination of tuberculomas from high grade gliomas and metastasis: a multimodal study. Neuroradiology. 2024;66(11):1979-1992. 39102087 10.1007/s00234-024-03435-7

[6] Eichorn FC, Polymicrobial brain abscesses: A complex condition with diagnostic and therapeutic challenges. J Neuropathol Exp Neurol. 2024;83(10):798-807. 38874452 10.1093/jnen/nlae058 PMC11413443

[7] Vibha D, A large cohort study of TB of the central nervous system: clinical outcomes. Int J Tuberc Lung Dis. 2022;26(10):989-991. 36163655 10.5588/ijtld.22.0229

[8] Heimer J, Comparison of the beta-hydroxybutyrate, glucose, and lactate concentrations derived from postmortem proton magnetic resonance spectroscopy and biochemical analysis for the diagnosis of fatal metabolic disorders. Int J Legal Med. 2020;134(2):603-612. 31900626 10.1007/s00414-019-02235-6

[9] Sulis G, Comparative Effectiveness of Regimens for Drug-Susceptible Tuberculous Meningitis in Children and Adolescents: A Systematic Review and Aggregate-Level Data Meta-Analysis. Open Forum Infect Dis. 2022;9(6):ofac108. 35673608 10.1093/ofid/ofac108 PMC9167638

